# Frequency of depression among patients with Type-I diabetes in a developing country, Pakistan

**DOI:** 10.12669/pjms.336.13911

**Published:** 2017

**Authors:** Musarrat Riaz, Nazish Imran, Asher Fawwad, Abdul Basit

**Affiliations:** 1 Musarrat Riaz, Baqai Institute of Diabetology and Endocrinology, Baqai Medical University, Karachi, Pakistan; 2 Nazish Imran, Department of Child and Family, King Edward Medical University, Lahore, Pakistan; 3 Asher Fawwad, Department of Research, Department of Biochemistry, Baqai Medical University, Karachi, Pakistan. Baqai Institute of Diabetology and Endocrinology, Baqai Medical University, Karachi, Pakistan; 4 Abdul Basit, Baqai Institute of Diabetology and Endocrinology, Baqai Medical University, Karachi, Pakistan

**Keywords:** Type-I diabetes, depression, Center for Epidemiologic Studies- Depression (CES-D)

## Abstract

**Objective:**

To determine the frequency of depressive symptoms among young people with Type-I diabetes.

**Methods:**

This cross sectional study was conducted at Baqai Institute of Diabetology & Endocrinology, Karachi, Pakistan from February to December 2015. All People aged between 12-20 years with Type-I diabetes for at least 1 year attending the OPD were included in the study. Information about participants’ demographic characteristics, co morbidities and Complications, current treatment and medications were obtained. Acylated hemoglobin (HbA1C) levels were checked in all People. The Center for Epidemiologic Studies Depression (CES-D) scale was used to assess the depressive symptoms in the study participants. A cut off value of ≥ 16 was used to screen for depression. SPSS 19 was used to analyze the results.

**Results:**

Out of 104 people with Type-I diabetes, depressive symptoms were observed in 44 (42.3%) participants. Depressive symptoms were more frequent in females (28/55, 50.9%). Depressed people had more episodes of DKA (11/44, 25%), hypoglycemia (12/44, 27.3%) or hospitalization (7/44, 15.9%) in the last six months which were not statistically significant.

**Conclusion:**

Depressive symptoms are quite common in people with Type-I diabetes. Health care professionals should consider screening youth with diabetes for depression regularly. Further large scale studies are needed to validate our findings.

## INTRODUCTION

Type-I diabetes mellitus (T1DM) is a complex chronic metabolic disorder affecting children and young adolescents.^1^ The management of T1DM is complex and involves multiple daily insulin injections with frequent monitoring of blood glucose, dietary restrictions with carbohydrate counting and a physically active lifestyle.^2^ Studies have demonstrated that this strict regimen with good glycemic control may prevent or delay various complications associated with T1DM.^3^

Children and young adolescent with T1DM are at increased risk of various psychosocial problems especially depression.^4^,^5^ Reported prevalence of depressive symptoms among adolescents with T1DM vary among various studies depending on the screening method used and the diagnostic criteria adopted^6^,^7^ and may vary from 2%-10%. In a study by Kovacs et al., the rates of psychiatric disorders were three times higher among people affected with T1DM than those without diabetes.^6^ In another study that followed youth with T1DM for 9 years, 42.4% of T1DM developed at least one episode of psychiatric disorder, most common being depression (27.5%) followed by anxiety (19.6%).^8^Studies have also demonstrated that depression in children is also associated with poor metabolic control^8^,^9^ retinopathy, female gender^10^ and children not living with both parents.^9^,^11^

These mental/psychological and behavioral health co- morbidities of diabetes are associated with poor adherence to treatment resulting in poor glycemic control,^12^ this in turn increases the risk for serious acute and chronic complications of diabetes resulting in blindness, chronic kidney diseases, stroke, amputations, and cardiovascular problems as well as decrease quality of life.^13^ Although numerous studies have shown an association of T1DM and depression among youth, most of them are conducted in developed countries where healthcare system is totally different from developing countries like Pakistan. Data from developing countries like Pakistan is scarce with only few studies focusing on this condition,^7^ hence this study was planned to find out the frequency of depressive symptoms among young people with T1DM and to explore the association between depressed mood and duration of diabetes, gender, glycemic control and hospitalization in the previous 6 months.

## METHODS

This cross sectional study was conducted at Baqai Institute of Diabetology & Endocrinology (BIDE), a tertiary care diabetes centre of Karachi, Pakistan. All People aged between 12-20 years with Type-I diabetes for at least one year attending the OPD were included in the study after obtaining inform consent. Ethical approval had been granted by the Institutional Review Board (IRB) of BIDE *(Ref: BIDE/IRB-/Dr.MRiaz /03/09/15/0074)*.

The trained interviewer approaches the parents (or legal guardian), for exclusion criteria, and invited families to enter a study of screening for psychological problems including depression and anxiety. Patients with known mental disorders or receiving psychotherapy were excluded. At the time of the study visit, informed consent was obtained. Information about participants demographic characteristics, co morbidities and complications, current treatment and medications were obtained by a trained diabetes educator. We considered participants to have co morbidities (kidney disease, celiac disease, hypertension, asthma, or polycystic ovarian syndrome) if they report having been diagnosed with any of these conditions. Participants or their parents/guardians were asked about the number of hospitalizations and Emergency visits and episodes of DKA and severe hypoglycemia that participants experienced in the previous 6 months. Sample size was estimated using the prevalence of depression among persons with Type-I diabetes reported in previous study.^14^ Using an estimated prevalence of 26%, 95% confidence intervals, and absolute precision of 1%, a sample size of 104 was determined.

Participants between 12-20 years of age were administered the Center for Epidemiologic Studies Depression (CES-D) scale by the interviewer in the local language Urdu after translation. Questions were explained to the child and parents, and the responses were documented.

The CES-D is a 20-item scale originally developed to measure depressive symptomatology in adult populations, and it has subsequently been used in studies of youth as young as 12 years of age. Respondents are asked how often they experienced 16 symptoms of depression and 4 symptoms of mental well-being during the previous week using a 4-point Liker scale (0, 1, 2, and 3). Their responses to each item are then added to create a single composite score, ranging from 0 to 60. A cut point to stratify depression severity as minimally (0 –15), mildly (16–23), and moderately/severely (24- 60) is used. Cronbach’s alpha was found to be 0.896.

### Statistical analysis

Data was analyzed using SPSS version 19.0. Frequencies of variables like gender, duration of diabetes, history of DKA, school abstinence etc. were calculated. Frequency of Depression was calculated by using a cut off value of ≥ 16.

## RESULTS

### Demographic & clinical characteristics

There were 104 participants in this study with mean age of 15.8 ± 3.1 years and included more females (55; 53%) than males (49; 47%). Mean duration of participant’s diabetes was 5.1 ± 4.0 years. History of DKA and hypoglycemia in the past six months was reported in 18.3% and 20.2% patients respectively. There were no significant differences between males and females in the sample with regards to any demographic and clinical variables. (P value>0.05). Table-I.

**Table-I:** Demographic & clinical characteristics of subjects categories by and gender.

*Variables*	*Overall*	*Male*	*Female*	*p-value*
n	104	49	55	-
Age (years)	15.8 ± 3.1	15.7 ± 3.1	15.9 ± 3.0	0.745
Weight (kg)	46.6 ± 13.2	48.5 ± 16.7	44.8 ± 8.3	0.236
Duration of diabetes (years)	5.1 ± 4.0	4.7 ± 3.7	5.4 ± 4.3	0.435
HbA1c (%)	10.3 ± 3.5	10.7 ± 3.1	10.0 ± 3.9	0.418
DKA episodes (past 6 months)				
Yes	19(18.3%)	7(14.3%)	12(21.8%)	0.321
No	85(81.7%)	42(85.7%)	43(78.2%)
Hypoglycemia episodes (past 6 months)				
Yes	21(20.2%)	11(22.4%)	10(18.2%)	0.588
No	83(79.8%)	38(77.6%)	45(81.8%)
Hospitalization (past 6 months)				
Yes	13(12.5%)	5(10.2%)	8(14.5%)	0.504
No	91(87.5%)	44(89.8%)	47(85.5%)
Reason of hospitalization				
DKA	9(8.7%)	3(6.1%)	6(10.9%)	0.553
Hypoglycemia	1(1%)	1(2%)	0(0%)	
Other	3(2.9%)	1(2%)	2(3.6%)
Co-morbid condition				
Yes	1(1.3%)	0(0%)	1(2.6%)	0.314
No	75(98.7%)	38(100%)	37(97.4%)
Co-morbid type				
Nephropathy	-	-	-	-
Retinopathy	1(1.3%)	-	1(1.3%)	
Neuropathy	-	-	-	
Others	-	-	-	
School absence days (past 6 months)	0.9 ± 2.9	0.4 ± 1.3	1.4 ± 3.8	0.189

Data presented as Mean±S.D or n (%) P-value < 0.05 is considered statistically significant

### Type-I Diabetes and Depression

In our sample, the frequency of depression as defined by CES (D) scores ≥ 16 was 44 (42.3%). Participants mean scores on CES-D was 16.2 ± 13.1 with females having significantly higher CES-D mean score (18.6 ± 13.7) compared to males (13.4 ± 12.0). (Table-II).

**Table-II:** Frequency of depression as per CES-D scores and categories by age and gender.

*Age Category*	*Male*	*Female*	*P-value*	*Overall*
n	49	55	-	104
CES-D mean score	13.4 ± 12.0	18.6 ± 13.7	0.043	16.2 ± 13.1
Normal (<16)	33 (67.3%)	27 (49.1%)	0.060	60 (57.7%)
Depressed (≥ 16)	16 (32.7%)	28 (50.9%)		44 (42.3%)
Age category				
12-15 years	5(31.2%)	9(32.1%)	0.951	14(31.8%)
16-20 years	11(68.8%)	19(67.9%)		30(68.2%)

Data presented as n (%), P-value < 0.05 is considered statistically significant, Cut point for depression is 16 or more

### Demographic & clinical characteristics comparison

There were no significant differences in people with or without depression in terms of demographic factors, self-reported school absenteeism, previous hospitalizations, comorbidities and neither disease related factors like duration of diabetes, hbA1c nor hypoglycemic and DKA episodes status differed significantly between the two groups. Table-III

**Table-III:** Demographic & clinical characteristics of subjects categories by depression status.

*Variables*	*Overall*	*Normal*	*Depressed*	*p-value*
n	104	60	44	-
Age (years)	15.8 ± 3.1	15.4 ± 3.0	16.4 ± 3.1	0.107
Weight (kg)	46.6 ± 13.2	47.4 ± 15.3	45.5 ± 9.9	0.540
Duration of diabetes (years)	5.1 ± 4.0	5.0 ± 4.3	5.1 ± 3.5	0.925
HbA1c (%)	10.3 ± 3.5	9.8 ± 3.9	10.9 ± 3.1	0.226
DKA episodes (past 6 months)				
Yes	19(18.3%)	8 (13.3%)	11 (25.0%)	0.128
No	85(81.7%)	52 (86.7%)	33 (75.0%)
Hypoglycemia episodes (past 6 months)				
Yes	21(20.2%)	9 (15.0%)	12 (27.3%)	0.123
No	83(79.8%)	51 (85.0%)	32 (72.7%)
Hospitalization (past 6 months)				
Yes	13(12.5%)	6 (10.0%)	7 (15.9%)	0.368
No	91(87.5%)	54 (90.0%)	37 (84.1%)
Reason of hospitalization				
DKA	9(8.7%)	4 (6.7%)	5 (11.4%)	0.530
Hypoglycemia	1(1%)	0 (0%)	1 (2.3%)	
Other	3(2.9%)	2 (3.3%)	1 (2.3%)
Co-morbid condition				
Yes	1(1.3%)	1 (2.3%)	0 (0%)	0.378
No	75(98.7%)	42 (97.7%)	33 (100%)
Co-morbid type				
Nephropathy	-	-	-	-
Retinopathy	1(100%)	1(100%)	-	
Neuropathy	-	-	-	
Others	-	-	-	
School absence days (past 6 months)	0.9 ± 2.9	0.7 ± 1.6	1.2 ± 3.7	0.443

Data presented as Mean±S.D or n (%) P-value < 0.05 is considered statistically significant.

## DISCUSSION

Purpose of current study was to examine frequency of depression in people with Type-I diabetes and its association with diabetes specific health outcomes. Results indicate that a substantial number of children in our sample (42.3%) reported clinically significant level of depressive symptoms. These results are in line with previous literature on this topic. A similar study done in Pakistan among diabetic patients aged 7-15 years observed depression in 33.7% patients.^7^ Depression was the most common psychiatric disorder in a 9 year follow up study of young people with Type-I diabetes.^6^ Rate of depression as high as 33% in people with Type-I diabetes was reported in another study.^9^ In another large-scale study of 2672 youth with Type-I diabetes, 14% had mild and 8.6% had moderate- severe depressed mood. Higher prevalence of depression in females was observed in the same study.^15^ Apart from higher prevalence, duration of depressive disorder was also noted to be longer in young people with diabetes compared to medically well young people.^6^ On the other hand, a fairly recent population based study results were in contrast to earlier reports with no evidence of increased psychopathology across a wide range of mental health measures in people with Type-I diabetes. Methodological discrepancies and better diabetes management reflecting in improvement in mental health have been considered as possible explanations for the results.^16^

Out of 44 participants having CES-D score of 16 or more depression was more common in female. This was probably because female patients are more vulnerable to family pressures after being diagnosed with diabetes as it is still considered a myth in our society. Type-I diabetes increases the challenges of childhood and in particular of adolescence, which is already a psychologically vulnerable period. Life style modifications, cognitive and emotional reactions to high and low blood sugar levels, child’s own perceptions of coping with diabetes, parental stress & wish of not being different from peers, may induce frustration and thus increase risk of psychiatric disturbances in young people.^17^

Although overall, life with Type-I diabetes has changed dramatically over the last decade, management of Type-I diabetes is still psychologically complex. There is evidence base for family centered interventions^18^ and coping skills training leading to improvement in psychological sequela in children with Type-Idiabetes.^19^

We found that young people with depression did not differ from young people without depression on any of the demographic and clinical parameters noted on current study. There are conflicting reports in literature about impact of depression on diabetes related parameters. Many studies suggest that increase in depressive symptoms are associated with many adverse diabetes specific health outcomes.^15^,^20^ Link of depression to poor metabolic control^8^, increase risk of retinopathy^21^ and increase hospitalization^22^ is documented. Martinez et al., found statistically significant correlations between a poor metabolic control (high levels of HbA1c) and depressive symptoms.^19^ A meta-analysis of depression and glycemic control in adults showed association of depression with poor glycemic control among adults with Type-I and Type-2 diabetes.^12^ On the other hand, authors of another study found an association between depression and glycemic control in only 2 of the 8 pediatric studies that they reviewed highlighting that impact of psychopathology & its relationship to metabolic control in Pediatric population does not yet appear clear.^22^ We also found no association between the frequency of depressed mood and the number of DKA and hypoglycemic episodes similar to SEARCH study^9^ nor of repeat hospitalizations^21^ among children and adolescents with diabetes.

Because of high prevalence of depression in young people with Type-I diabetes and evidence of possible adverse impact in clinical outcomes, American Diabetes association has recommended regular annual screening for depression in children age 10 or older with Type-I diabetes.^23^

### Limitations of the Study

The present study is unique in that it emphasizes the holistic practice of medicine and biopsychosocial model of care. However, some limitations of the study must be acknowledged. The cross-sectional nature of study means no temporal associations of depression and possible long term adverse outcomes on study parameters can be determined. Data collection from one center may limit generalizability of findings. Furthermore, we assessed only for depressive symptomatology. It would help to do a more comprehensive assessment of possible psychopathology in this vulnerable group like anxiety, self-esteem issues, and sleep related difficulties.

## CONCLUSION

Depressive symptoms are quite common in people with Type-I diabetes, therefore doctors and other health care professionals, including diabetes educators, should consider screening youth with diabetes for depression regularly. Future research is needed to address other relevant aspects related to young people with type I diabetes including their quality of life, parental stress, perceptions regarding illness & use of coping strategies.
